# Supplementary material for: Cytosolic aspartate aminotransferase moonlights as a ribosome-binding modulator of Gcn2 activity during oxidative stress
Source: eLife. 2022 May 27;11:e73466. doi: 10.7554/eLife.73466 (PMC9191892; doi:10.7554/eLife.73466)

SOURCE DATA

Figure 5a – western blot images

Anti-Sui2/eIF2α

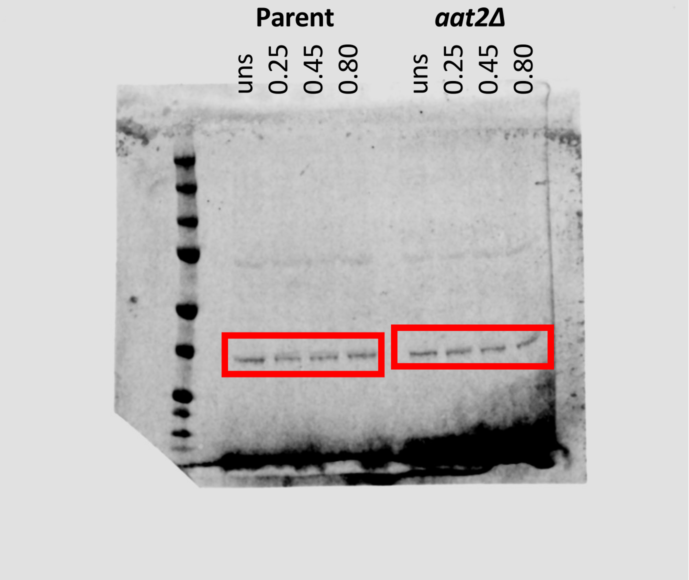

Anti-phospho-Sui2/eIF2α

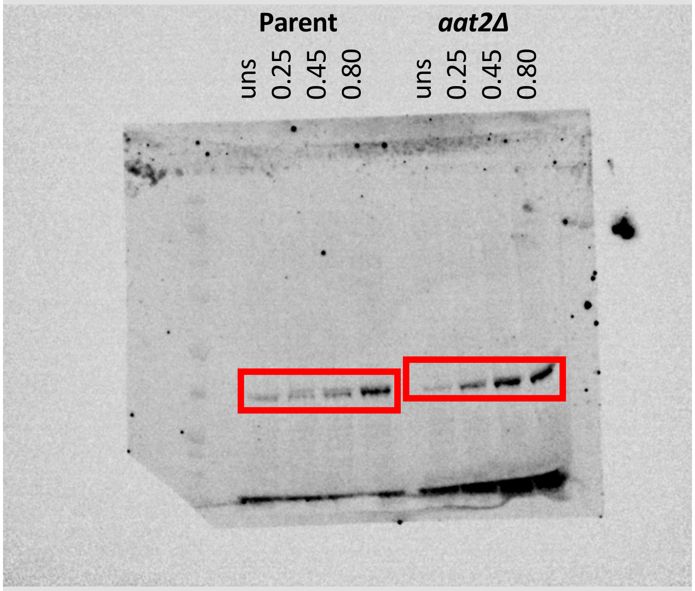

Anti-Pab1

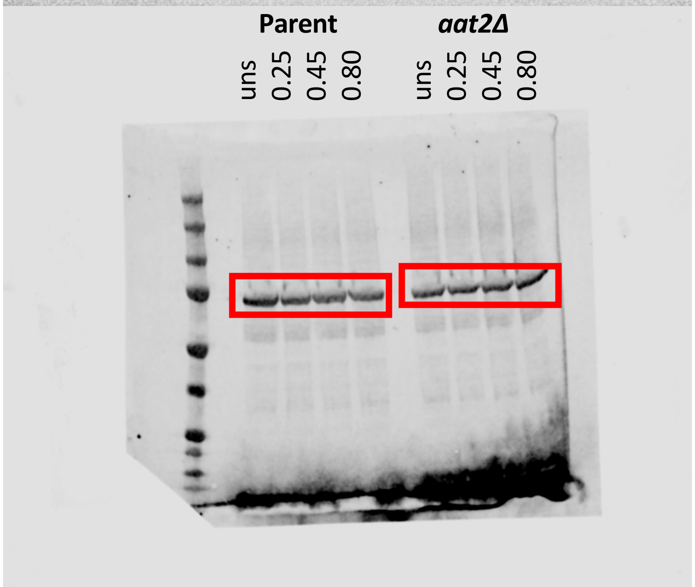

SOURCE DATA

Figure 6a – western blot images

Anti-Pab1

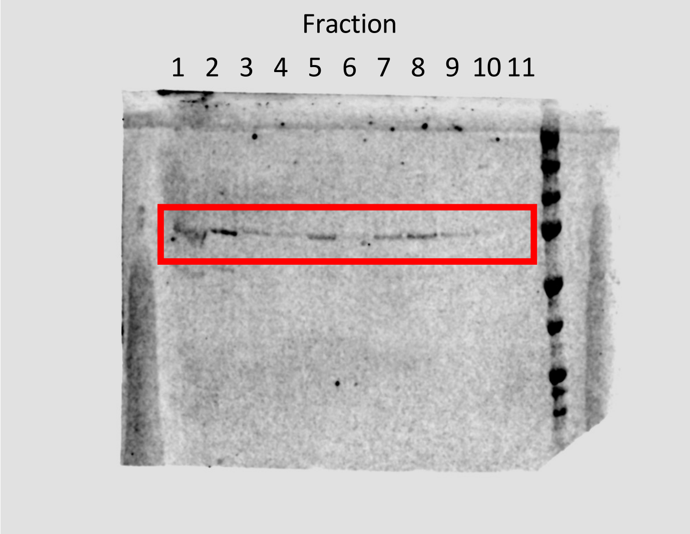

Anti-Scp160

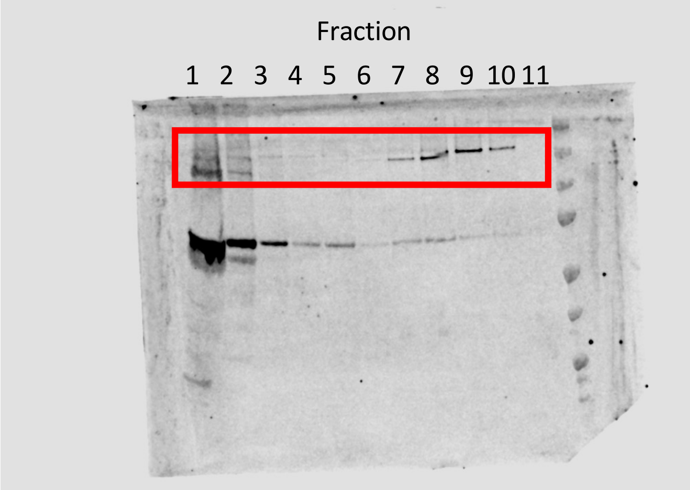

Anti-protein A (Aat2-TAP)

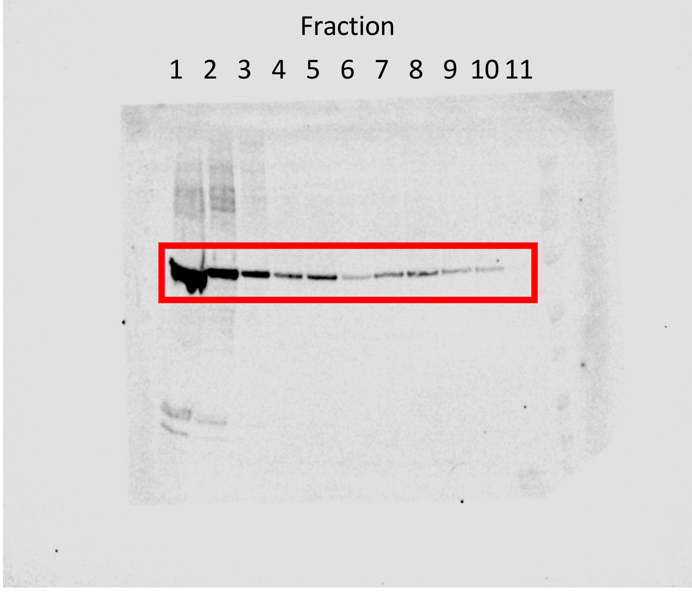

SOURCE DATA

Figure 6b (left panels, untreated) – western blot images

Anti-Pab1

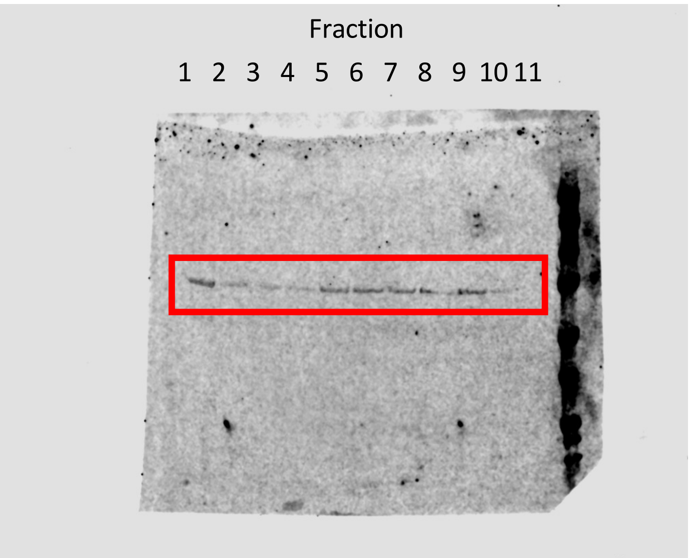

Anti-Scp160

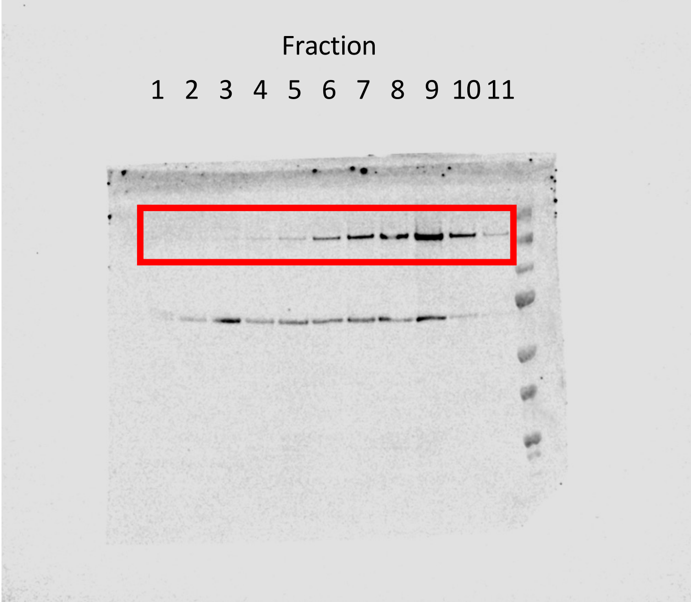

Anti-protein A (Aat2-TAP)

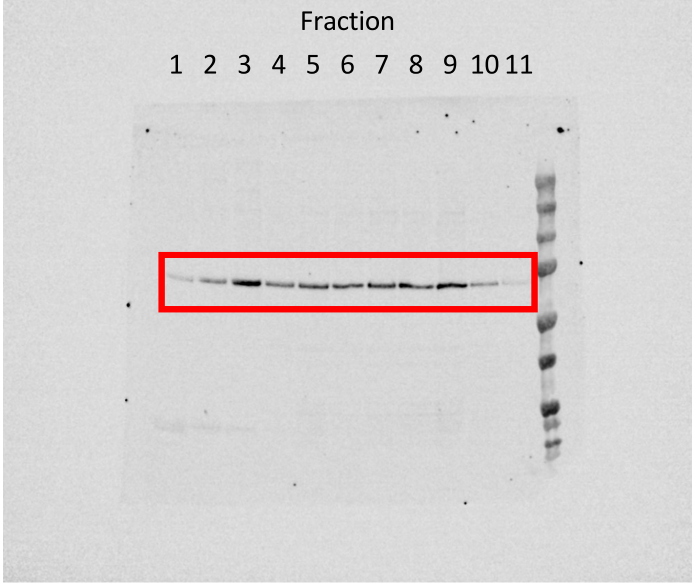

SOURCE DATA

Figure 6b (right panels, +RNase) – western blot images

Anti-Pab1

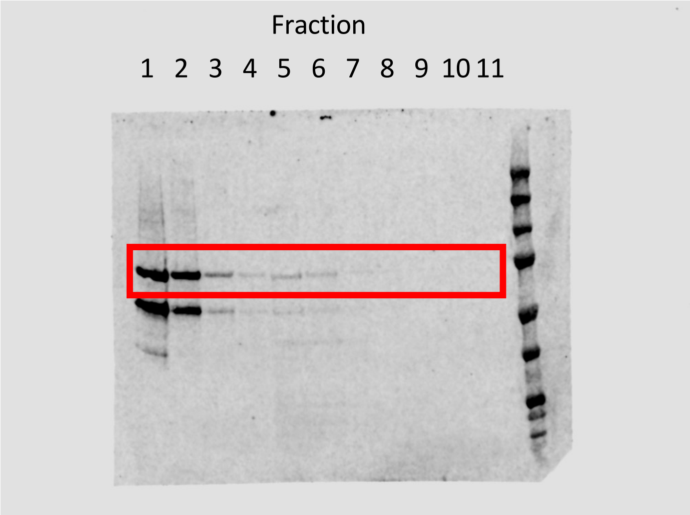

Anti-Scp160

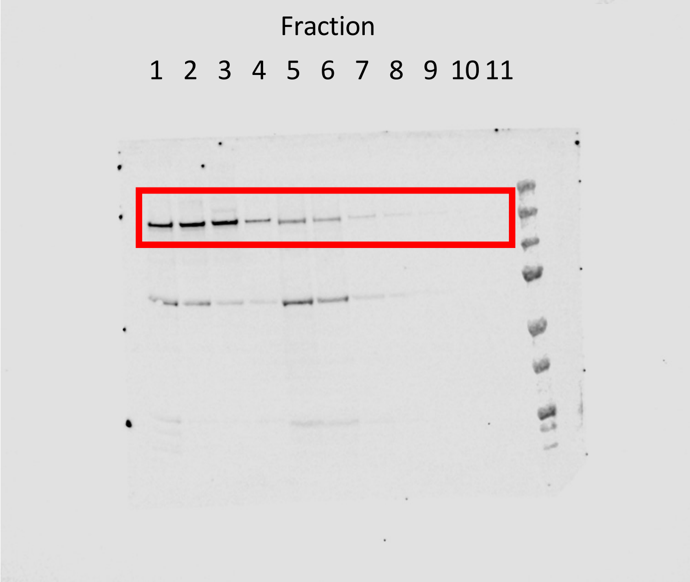

Anti-protein A (Aat2-TAP)

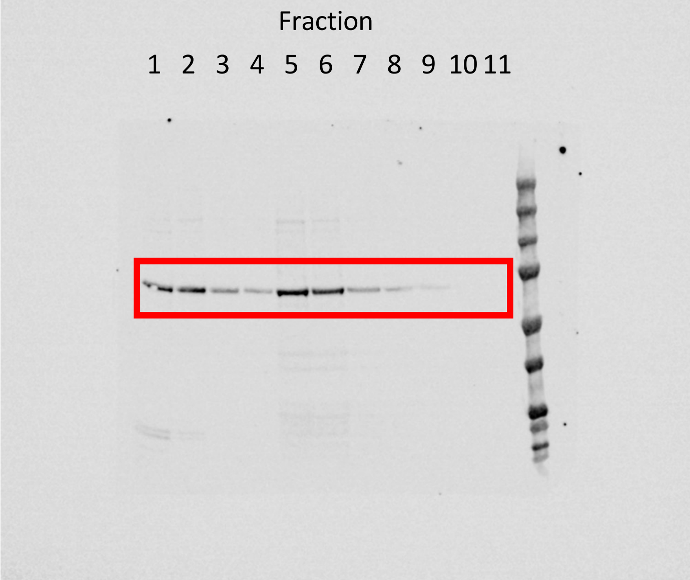

SOURCE DATA

Figure 6c – western blot images

Anti-Pab1

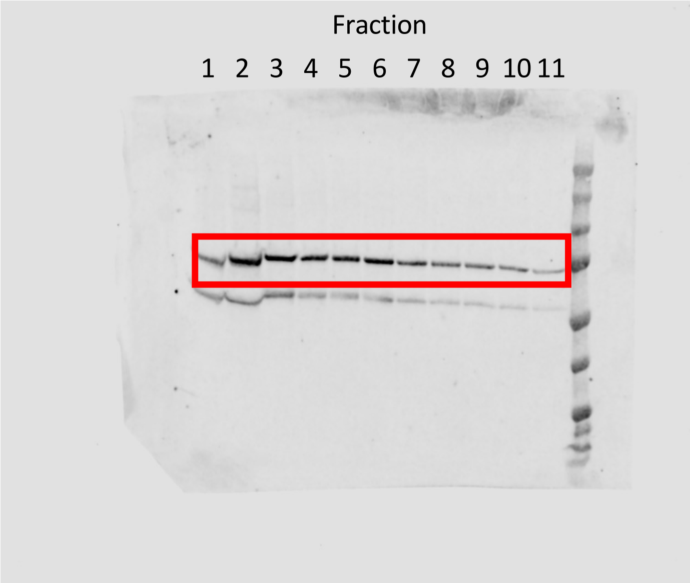

Anti-Scp160

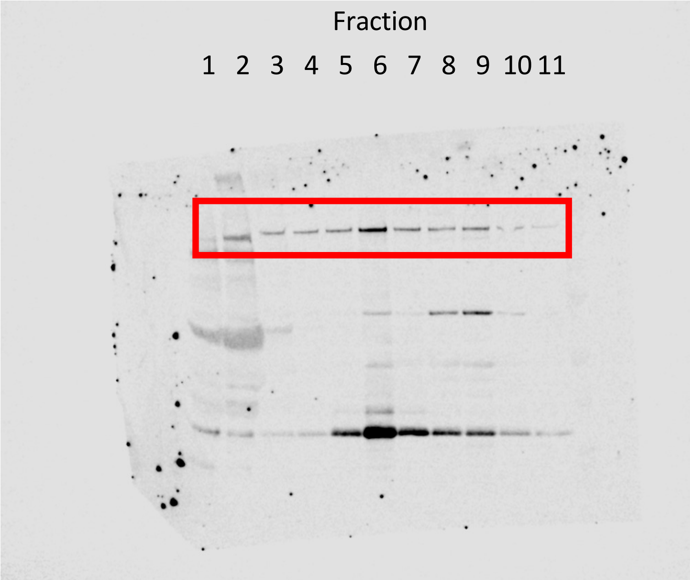

Anti-protein A (Aat2-TAP)

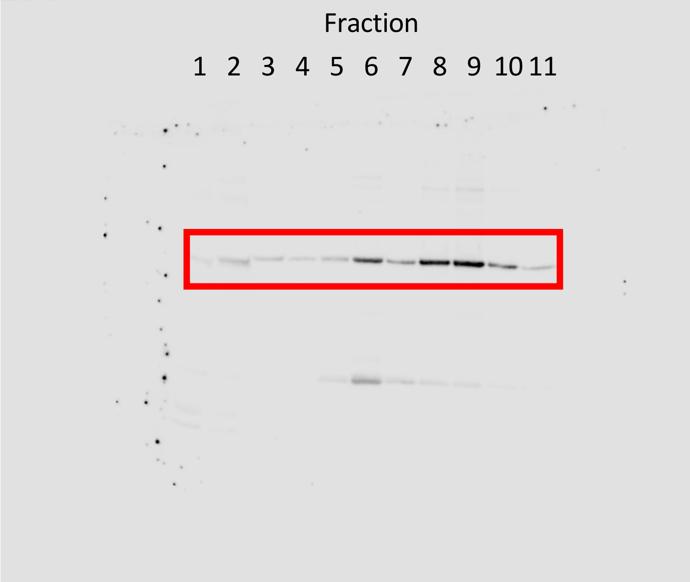

SOURCE DATA

Figure 6c – western blot images

Anti-Rpl35

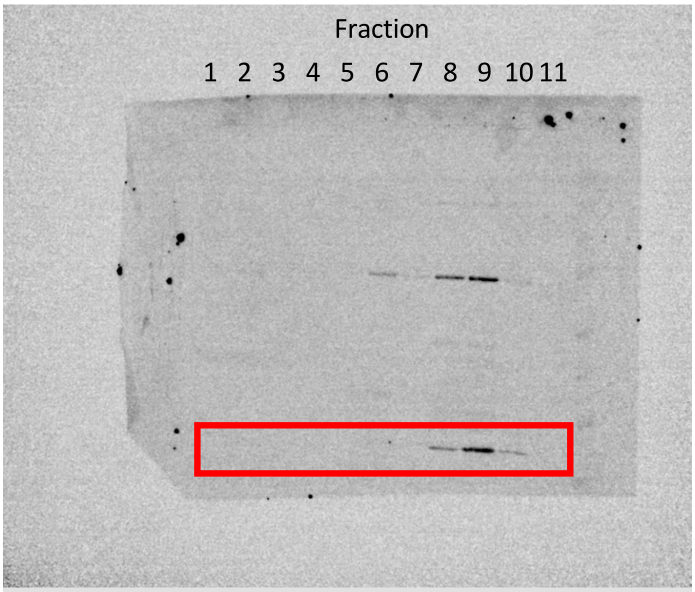

Anti-Rps3

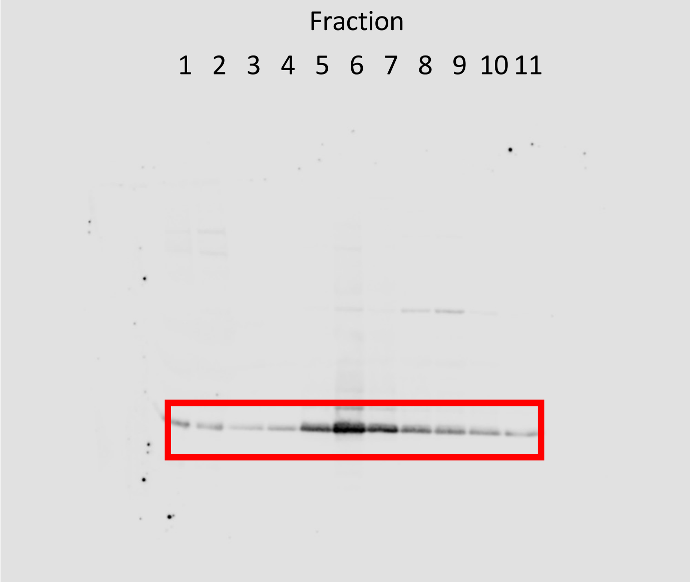

SOURCE DATA

Figure 7b (left panels, unstressed, SM) – western blot images

Anti-Pab1

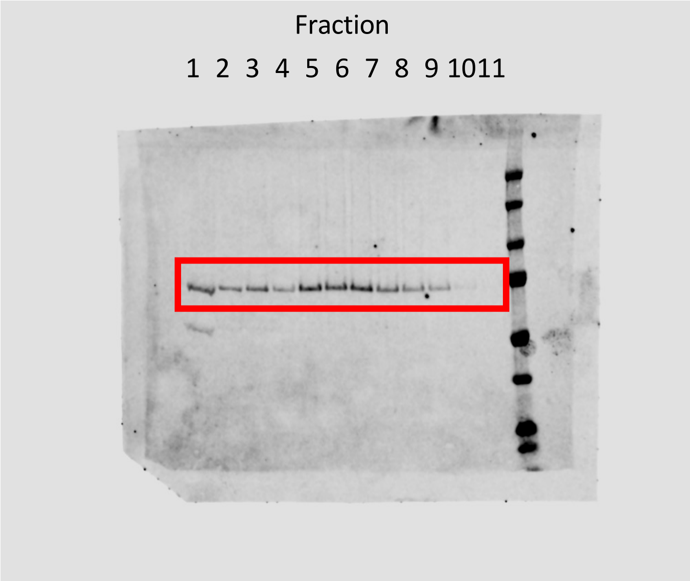

Anti-protein A (Aat2-TAP)

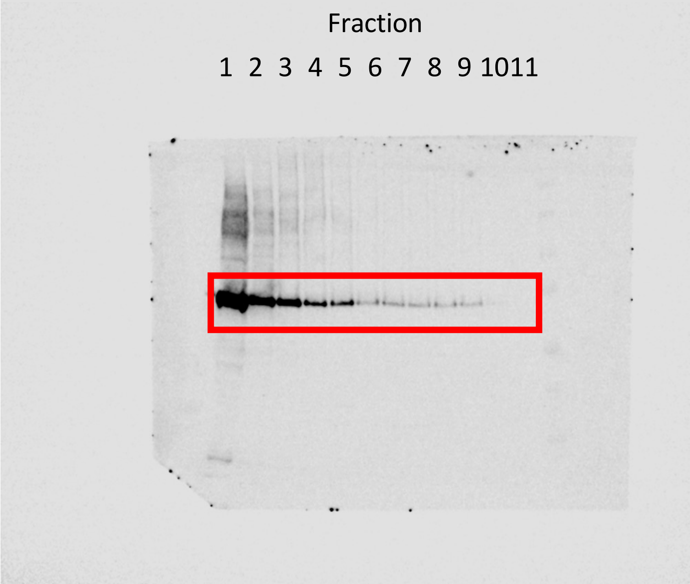

SOURCE DATA

Figure 7b (left panels, unstressed, K255E) – western blot imag

Anti-Pab1

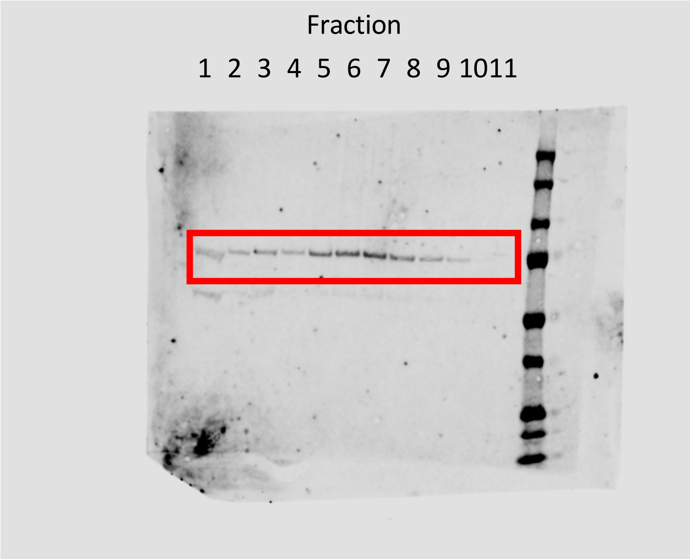

Anti-protein A (Aat2-TAP)

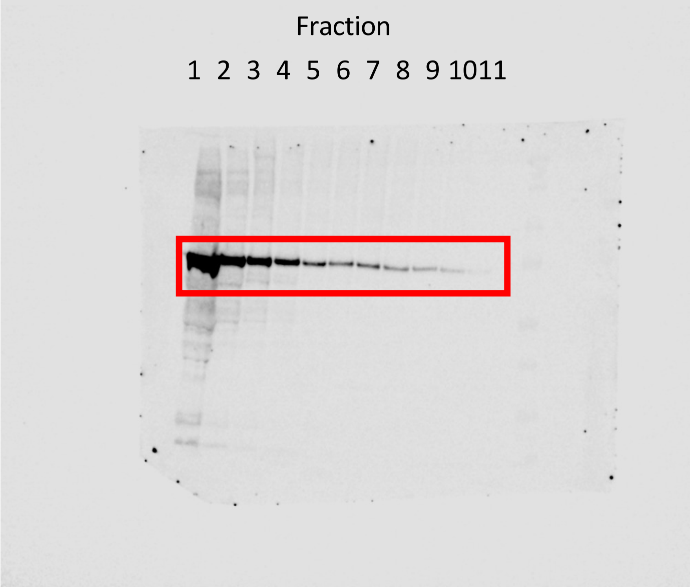

SOURCE DATA

Figure 7b (left panels, unstressed, R387E) – western blot imag

Anti-Pab1

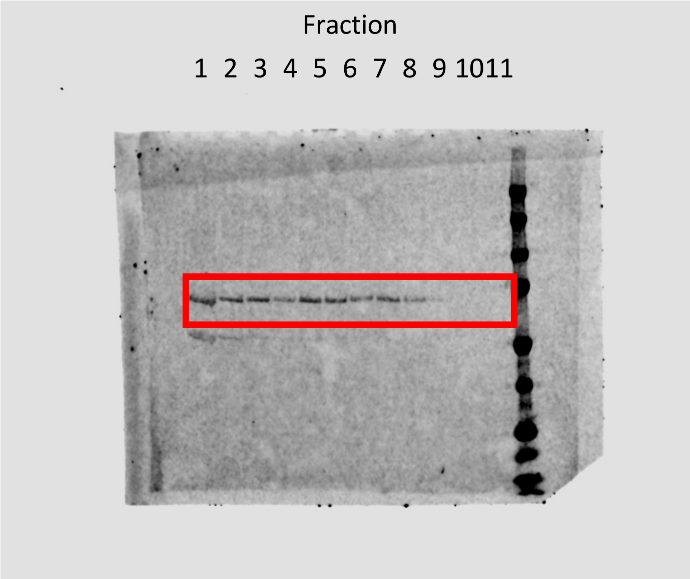

Anti-protein A (Aat2-TAP)

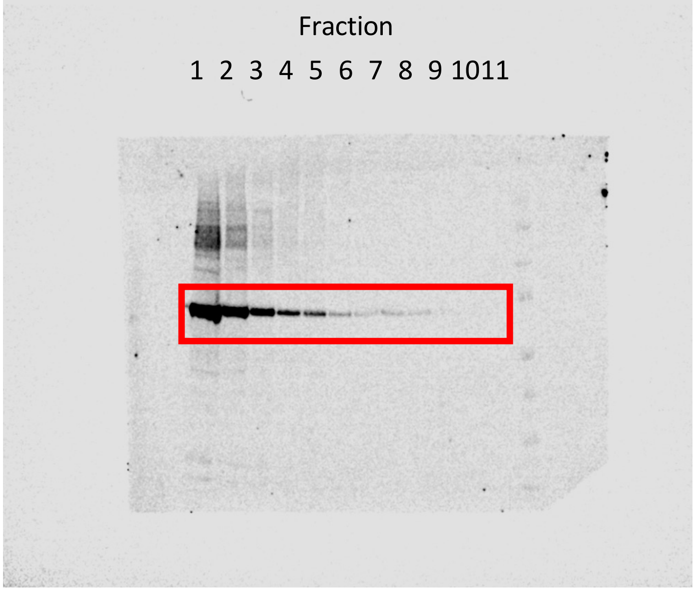

SOURCE DATA

Figure 7b (right panels, +H2O2, SM) – western blot images

Anti-Pab1

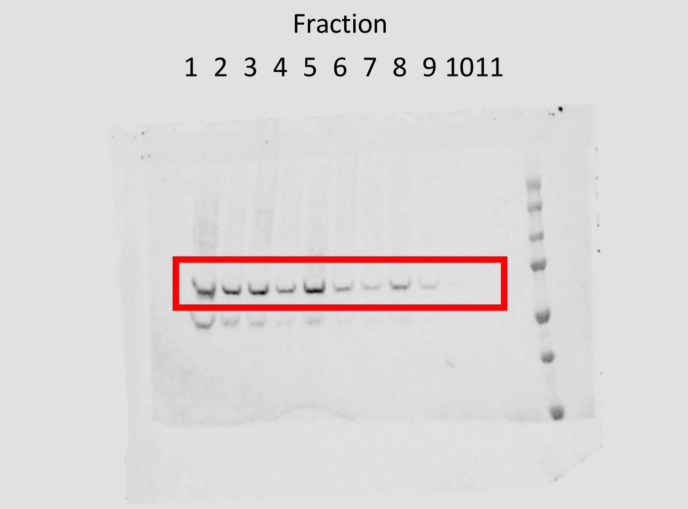

Anti-protein A (Aat2-TAP)

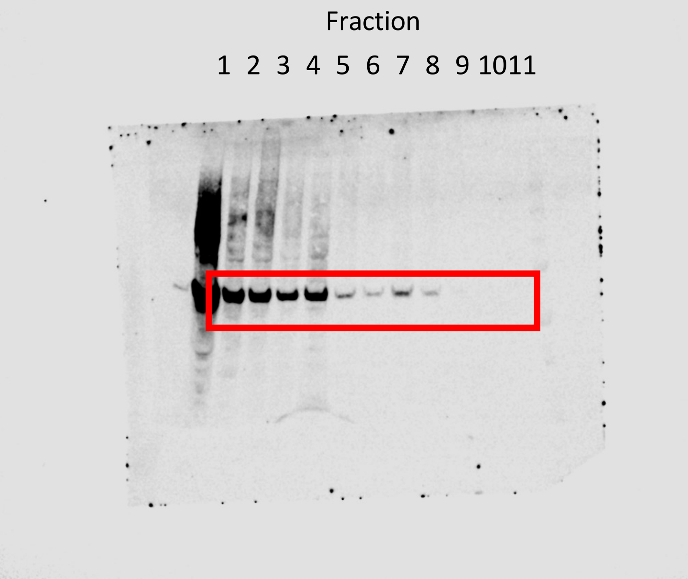

SOURCE DATA

Figure 7b (right panels, +H2O2, K255E) – western blot images

Anti-Pab1

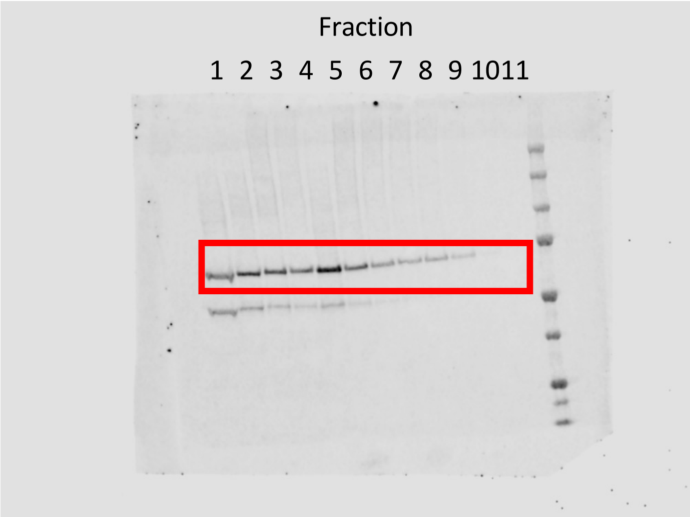

Anti-protein A (Aat2-TAP)

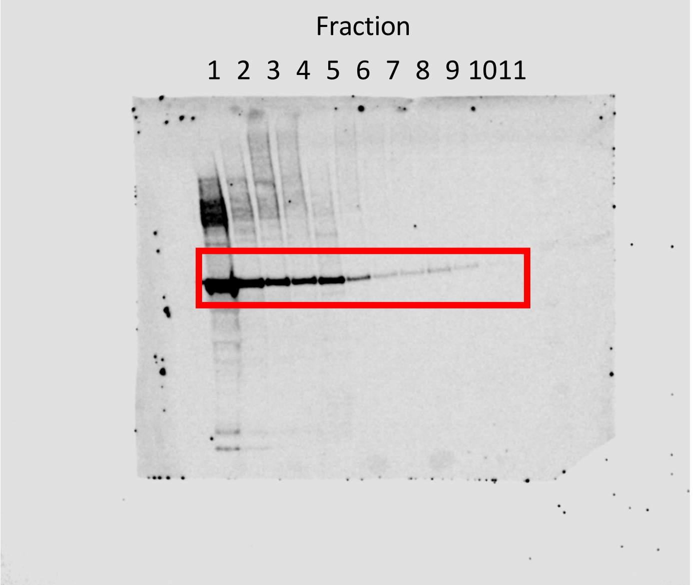

SOURCE DATA

Figure 7b (right panels, +H2O2, R387E) – western blot images

Anti-Pab1

Anti-protein A (Aat2-TAP)

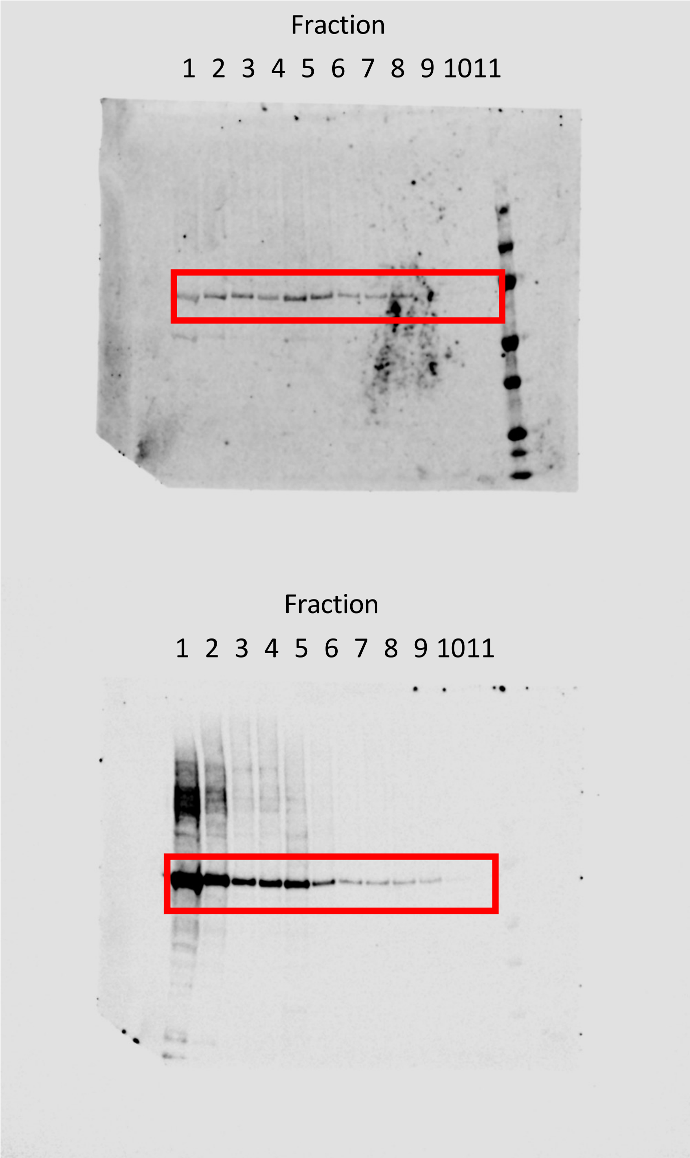

SOURCE DATA

Figure 7d – western blot images

Three (parent, *aat2Δ*) or four (K255E, R387E) replicates were performed and loaded on to separate gels. Each set of samples is an independent replicate, except for the Parent samples on gels 1 and 3, which are the same.

The Parent +0.45 mM H2O2 sample from gel 1 was loaded on each gel so that bands could be normalised to a common sample (lane N on all gels).

Representative samples shown in figures highlighted in red, all others in orange.

SOURCE DATA

Figure 7d – western blot images (gel 1)

Anti-Sui2/eIF2α

N = normaliser. Same sample (Parent +0.45 mM H2O2) loaded on each gel for normalisation

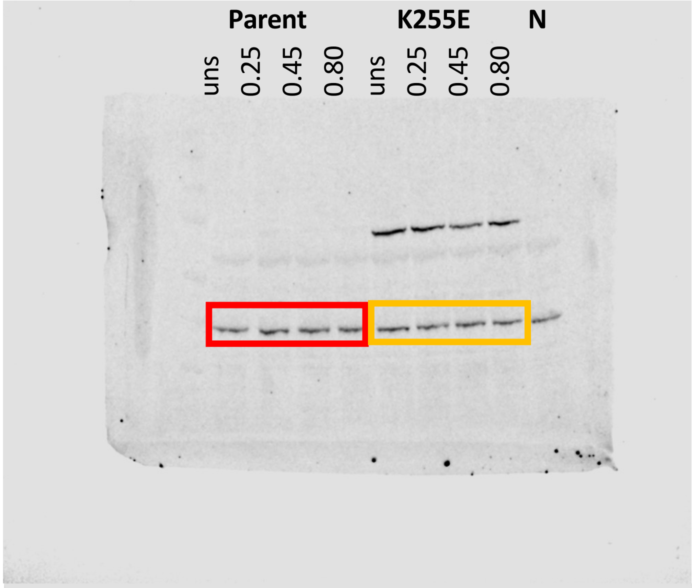

Anti-phospho-Sui2/eIF2α

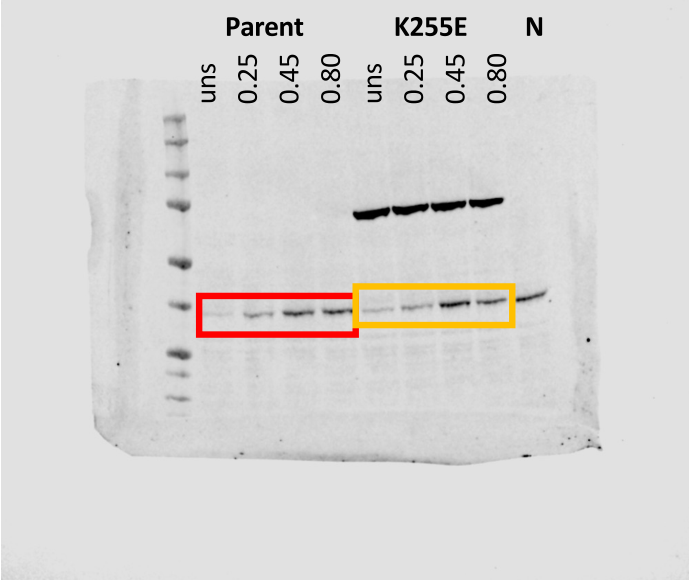

Anti-Pab1

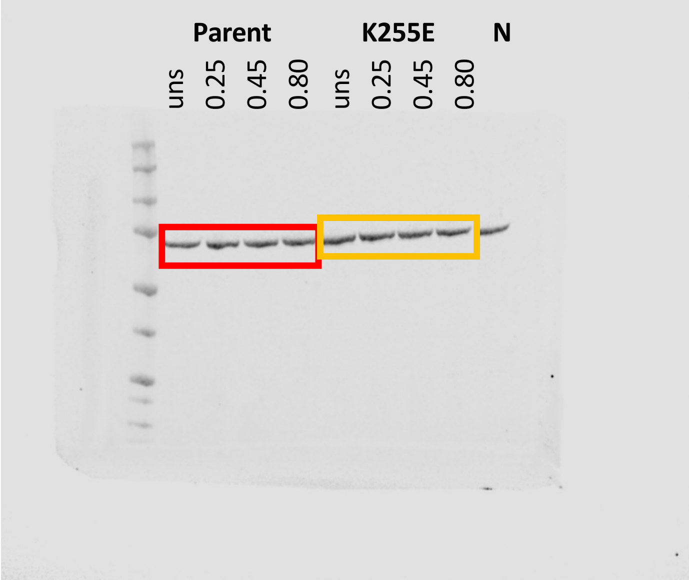

SOURCE DATA

Figure 7d – western blot images (gel 2)

Anti-Sui2/eIF2α

N = normaliser. Same sample (Parent +0.45 mM H2O2) loaded on each gel for normalisation

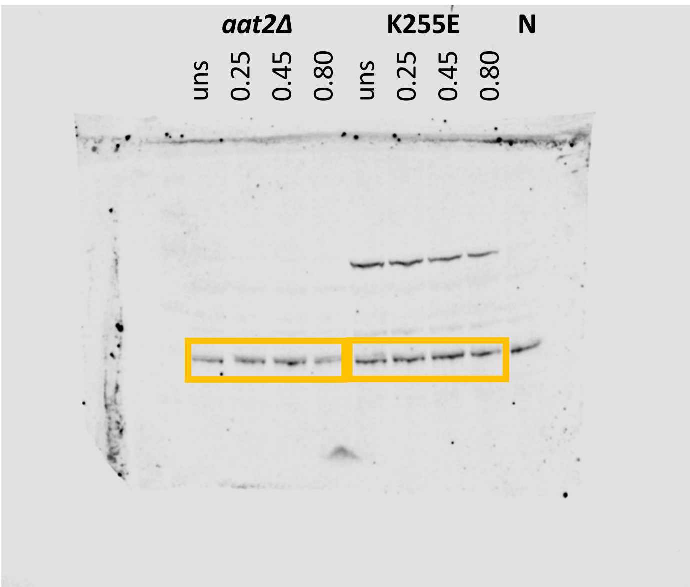

Anti-phospho-Sui2/eIF2α

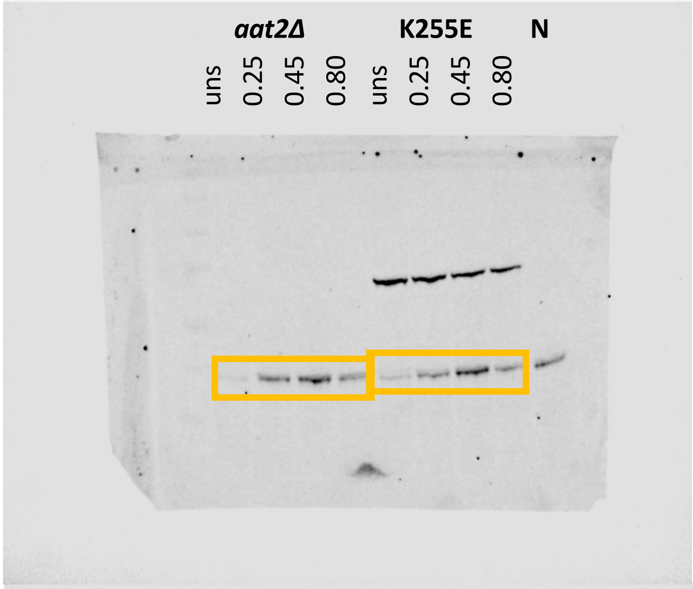

Anti-Pab1

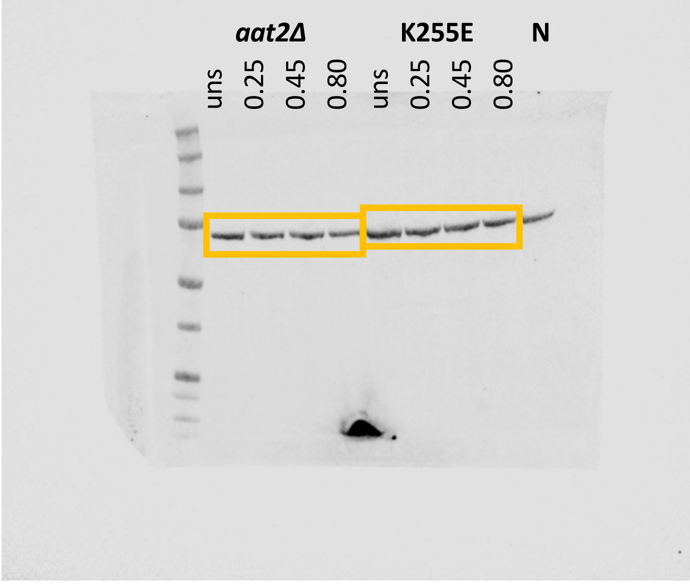

SOURCE DATA

Figure 7d – western blot images (gel 3)

Anti-Sui2/eIF2α

N = normaliser. Same sample (Parent +0.45 mM H2O2) loaded on each gel for normalisation

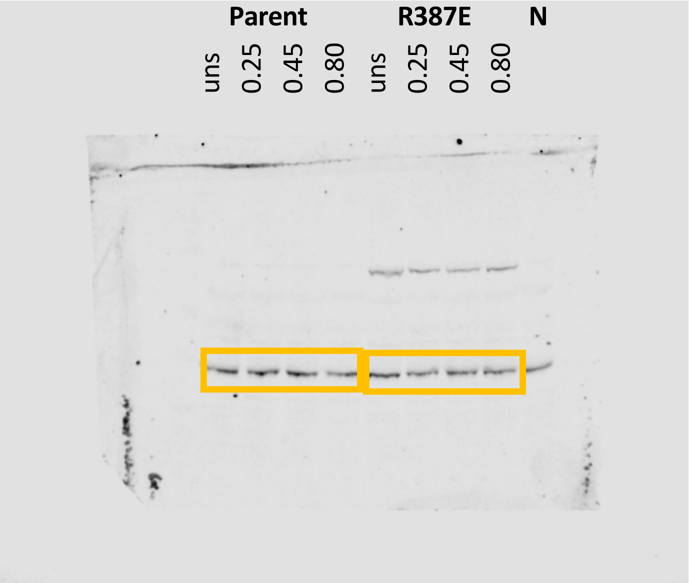

Anti-phospho-Sui2/eIF2α

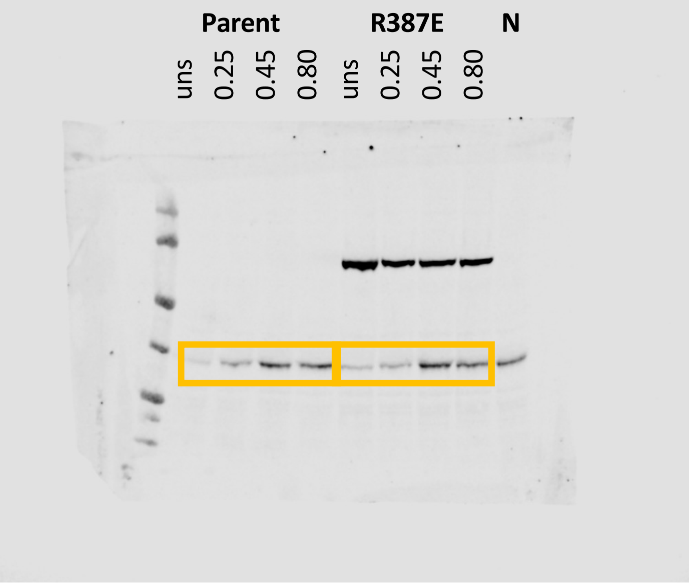

Anti-Pab1

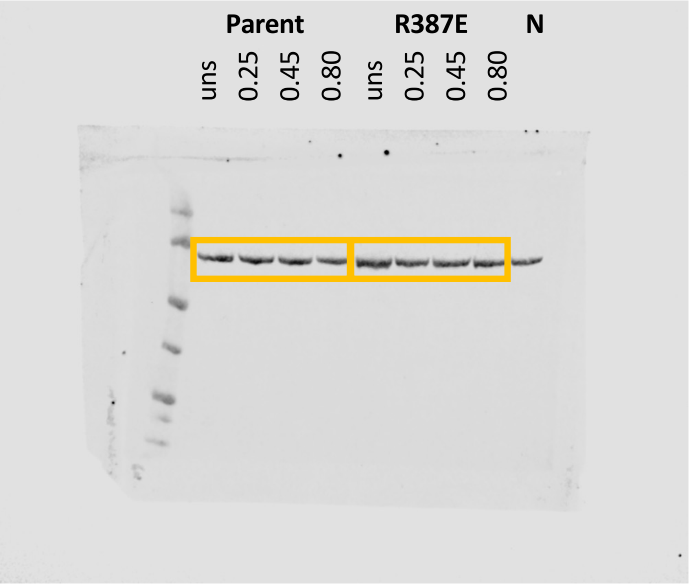

SOURCE DATA

Figure 7d – western blot images (gel 4)

Anti-Sui2/eIF2α

N = normaliser. Same sample (Parent +0.45 mM H2O2) loaded on each gel for normalisation

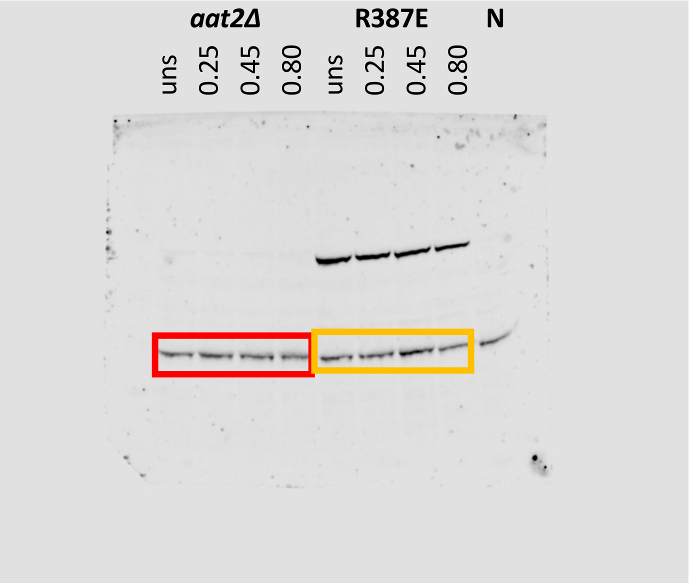

Anti-phospho-Sui2/eIF2α

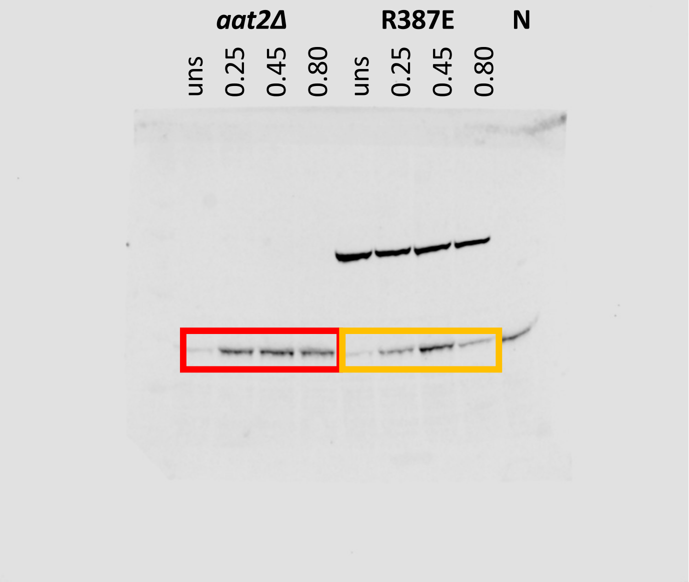

Anti-Pab1

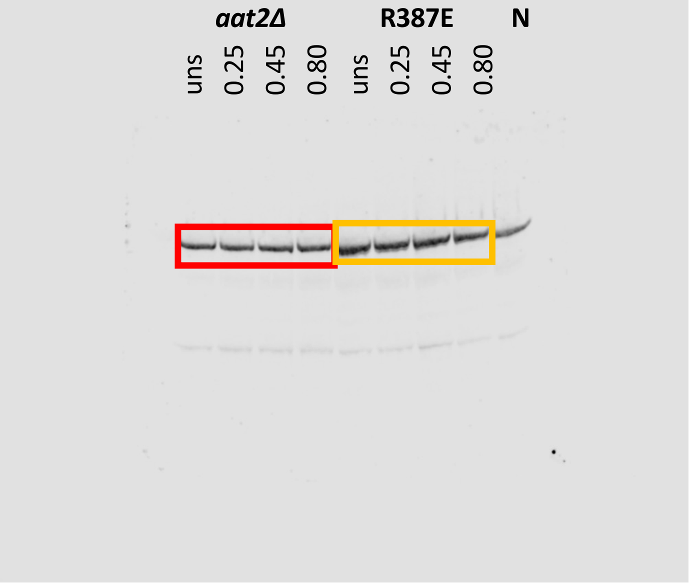

SOURCE DATA

Figure 7d – western blot images (gel 5)

Anti-Sui2/eIF2α

N = normaliser. Same sample (Parent +0.45 mM H2O2) loaded on each gel for normalisation

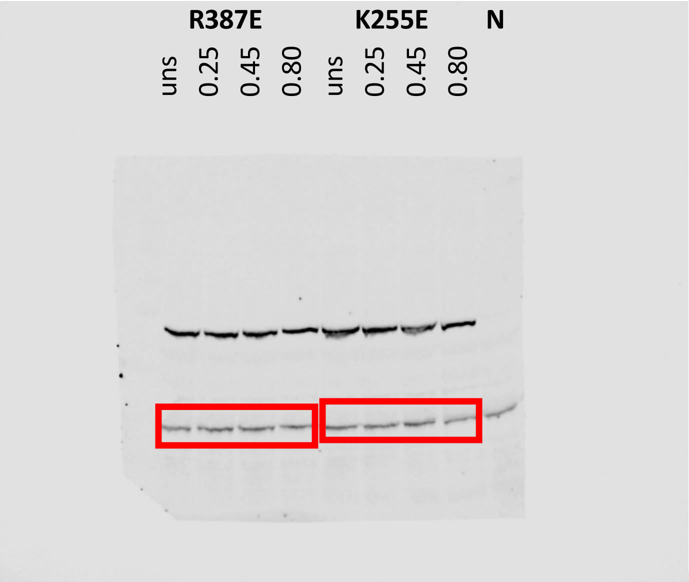

Anti-phospho-Sui2/eIF2α

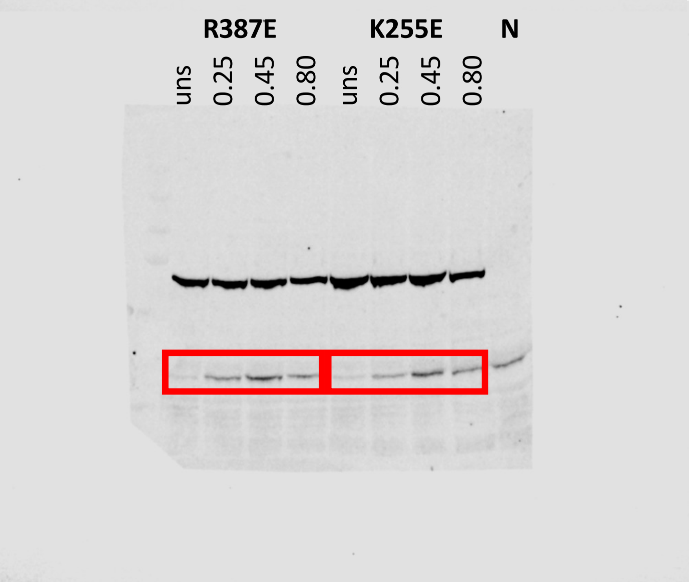

Anti-Pab1

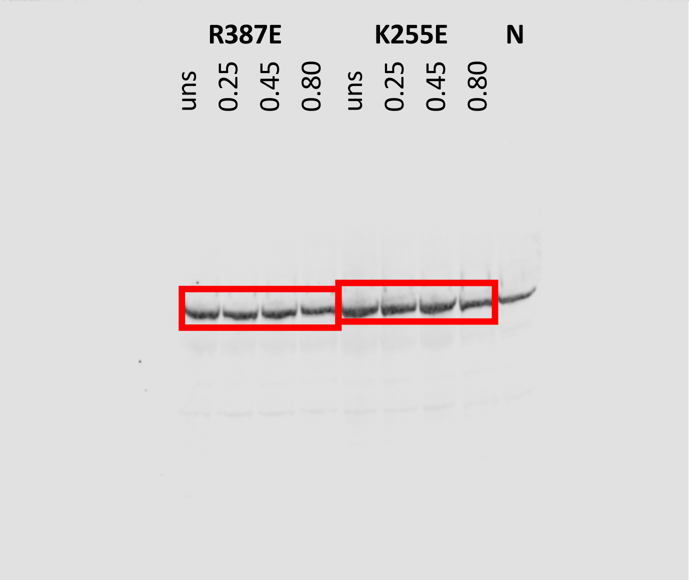

SOURCE DATA

Figure 7d – western blot images (gel 6)

Anti-Sui2/eIF2α

N = normaliser. Same sample (Parent +0.45 mM H2O2) loaded on each gel for normalisation

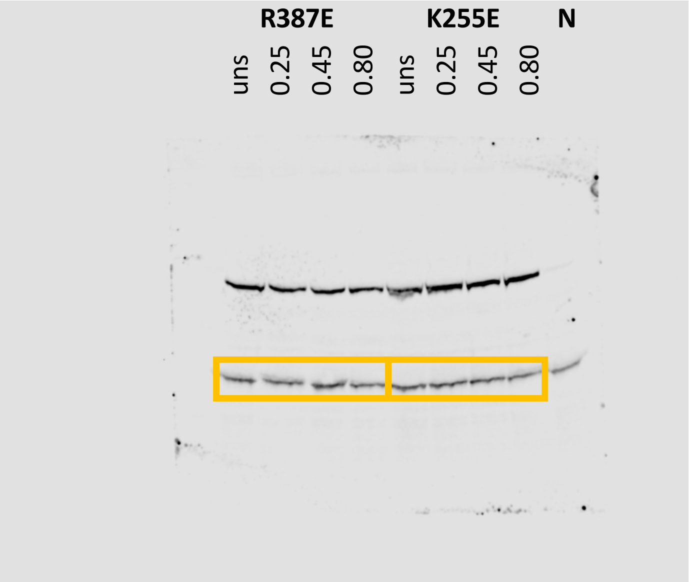

Anti-phospho-Sui2/eIF2α

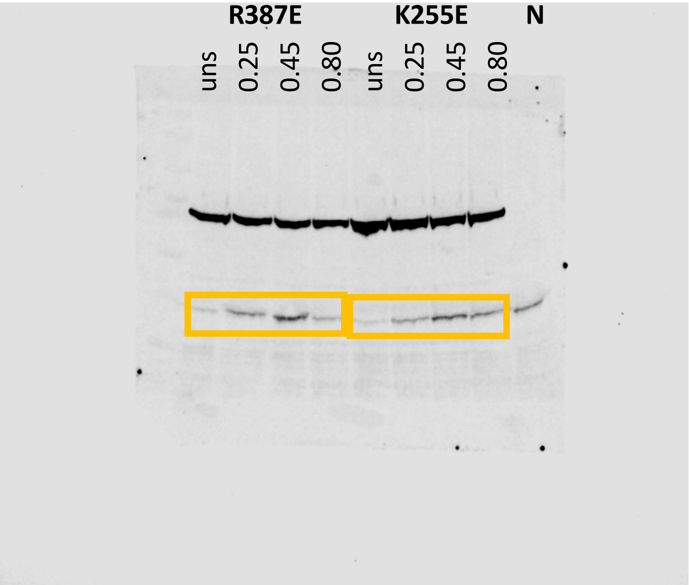

Anti-Pab1

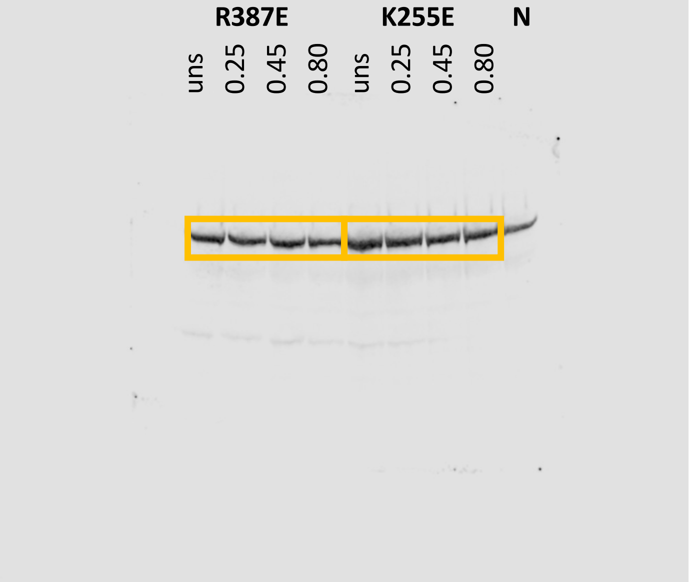

SOURCE DATA

Figure 7d – western blot images (gel 7)

Anti-Sui2/eIF2α

N2 = normaliser. Parent +0.80 mM H2O2 loaded here and on gel 8 for normalisation

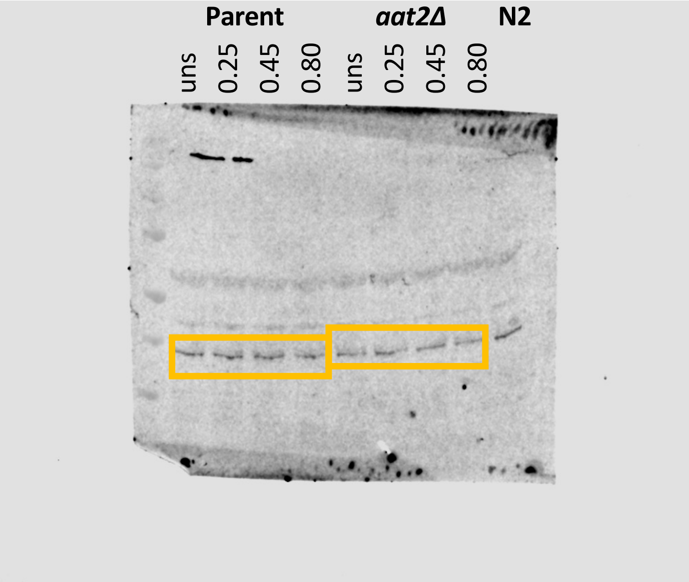

Anti-phospho-Sui2/eIF2α

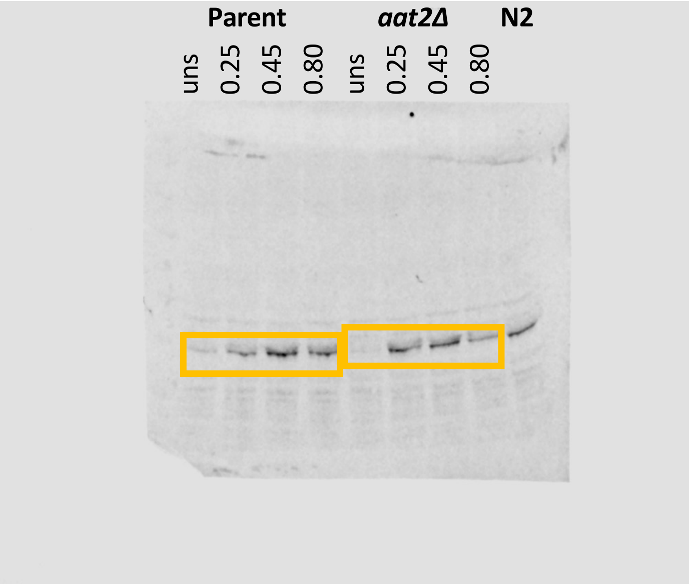

SOURCE DATA

Figure 7d – western blot images (gel 8)

Anti-Sui2/eIF2α

N (Parent +0.45 mM H2O2) and N2 (Parent +0.80 mM H2O2) = normalisers

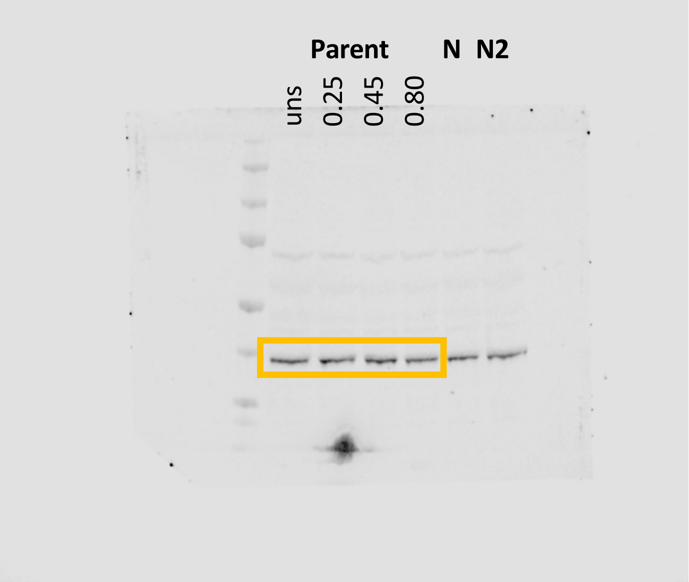

Anti-phospho-Sui2/eIF2α

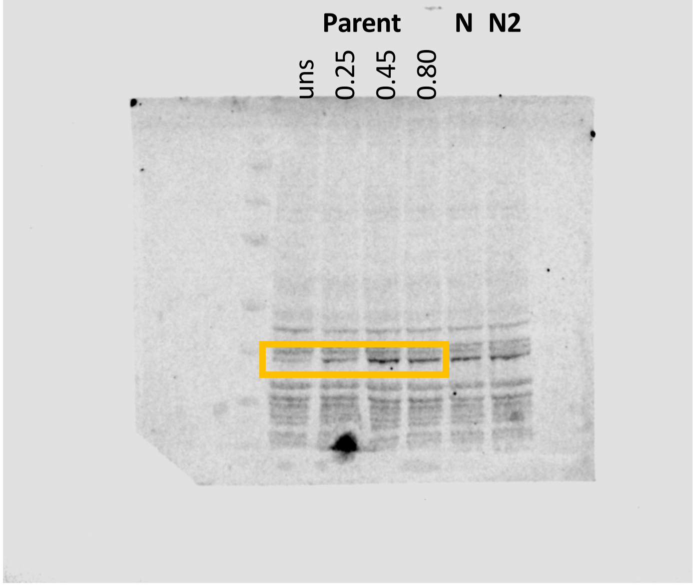

Supplement: Source data 1. [file elife-73466-data1.pdf]
